# GIP receptor agonism improves dyslipidemia and atherosclerosis independently of body weight loss in preclinical mouse model for cardio-metabolic disease

**DOI:** 10.1186/s12933-023-01940-2

**Published:** 2023-08-17

**Authors:** Stephan Sachs, Anna Götz, Brian Finan, Annette Feuchtinger, Richard D. DiMarchi, Yvonne Döring, Christian Weber, Matthias H. Tschöp, Timo D. Müller, Susanna M. Hofmann

**Affiliations:** 1 Institute for Diabetes and Regeneration, Helmholtz Diabetes Center at Helmholtz Zentrum München, German Research Center for Environmental Health (GmbH), 85764 Neuherberg, Germany; 2 Institute for Diabetes and Obesity, Division of Metabolic Diseases, Helmholtz Diabetes Center at Helmholtz Centre Munich, Munich, Germany; 3 Technische Universität München, 80333 Munich, Germany; 4 Novo Nordisk Research Center Indianapolis, Indianapolis, IN USA; 5 Research Unit Analytical Pathology, Helmholtz Center Munich, 85764 Neuherberg, Germany; 6 Department of Chemistry, Indiana University, Bloomington, IN USA; 7 Department of Angiology, Swiss Cardiovascular Center, Inselspital, Bern University Hospital, University of Bern, Bern, Switzerland; 8 Institute for Cardiovascular Prevention (IPEK), Ludwig-Maximilians-University Munich, Munich, Germany; 9 DZHK (German Centre for Cardiovascular Research), Partner Site Munich Heart Alliance, Munich, Germany; 10 Department of Biochemistry, Cardiovascular Research Institute Maastricht (CARIM), Maastricht University, Maastricht, the Netherlands; 11 Munich Cluster for Systems Neurology (SyNergy), Munich, Germany; 12 German Center for Diabetes Research (DZD), 85764 Neuherberg, Germany; 13 Department of Medicine IV, University Hospital, LMU Munich, Munich, Germany

**Keywords:** GIP agonist, acyl-GIP, Obesity, Dyslipidemia, Atherosclerosis, Cardiometabolic disease, Mice

## Abstract

**Background:**

Agonism at the receptor for the glucose-dependent insulinotropic polypeptide (GIPR) is a key component of the novel unimolecular GIPR:GLP-1R co-agonists, which are among the most promising drugs in clinical development for the treatment of obesity and type 2 diabetes. The therapeutic effect of chronic GIPR agonism to treat dyslipidemia and thus to reduce the cardiovascular disease risk independently of body weight loss has not been explored yet.

**Methods:**

After 8 weeks on western diet, LDL receptor knockout (LDLR-/-) male mice were treated with daily subcutaneous injections of long-acting acylated GIP analog (acyl-GIP; 10nmol/kg body weight) for 28 days. Body weight, food intake, whole-body composition were monitored throughout the study. Fasting blood glucose and intraperitoneal glucose tolerance test (ipGTT) were determined on day 21 of the study. Circulating lipid levels, lipoprotein profiles and atherosclerotic lesion size was assessed at the end of the study. Acyl-GIP effects on fat depots were determined by histology and transcriptomics.

**Results:**

Herein we found that treatment with acyl-GIP reduced dyslipidemia and atherogenesis in male LDLR-/- mice. Acyl-GIP administration resulted in smaller adipocytes within the inguinal fat depot and RNAseq analysis of the latter revealed that acyl-GIP may improve dyslipidemia by directly modulating lipid metabolism in this fat depot.

**Conclusions:**

This study identified an unanticipated efficacy of chronic GIPR agonism to improve dyslipidemia and cardiovascular disease independently of body weight loss, indicating that treatment with acyl-GIP may be a novel approach to alleviate cardiometabolic disease.

**Supplementary Information:**

The online version contains supplementary material available at 10.1186/s12933-023-01940-2.

## Background

Alterations in lipid and cholesterol metabolism are major risk factors for the development of cardiovascular disease (CVD) in patients with obesity and type-2 diabetes (T2D). Albeit best known for its ability to enhance glucose-stimulated insulin secretion, the glucose-dependent insulinotropic polypeptide (GIP) also stimulates white adipose tissue (WAT) lipid disposal and reduces inflammation in the brain and WAT [1]. Unimolecular co-agonists at the receptors for GIP and the glucagon-like peptide-1 (GLP-1) are among the most promising drugs in clinical development for the treatment of obesity and diabetes [2]. Notably, GLP-1/GIP co-agonists not only reduce body weight and improve glucose metabolism with greater efficacy relative to GLP-1 receptor (GLP-1R) agonism in preclinical [3] and clinical studies [2], but also outperform GLP-1R monotherapy in reducing triglyceride and cholesterol levels [4]. However, the therapeutic effect of GIP receptor (GIPR) agonism to treat dyslipidemia and reduce CVD-risk is not well defined yet and thus subject of intense ongoing investigations. Particularly, it warrants clarification whether GIP may even improve lipid metabolism independent of its ability to reduce obesity and hyperglycemia.

GIP is secreted from enteroendocrine K-cells especially in response to dietary lipids and glucose. The biological function of GIP to potentiate glucose-dependent beta cell insulin secretion (incretin effect) is well established [for review see 5]. GIP’s extra-pancreatic actions are less known and especially its pro- or anti-obesogenic effects are controversially discussed [6]. In brief, GIP might have an indirect role in atherosclerosis, via the regulation of macrophage-driven inflammation and foam cell formation, vascular smooth muscle cell proliferation and arterial remodelling. However, it has also been shown that increased plasma levels of GIP are associated with atherosclerosis in humans [7]. Recent success of GIP as add-on therapy to glucagon-like peptide 1 (GLP-1) in unimolecular dual incretins to glucose and body weight improvements in pre-clinical and clinical studies indicate GIP-dependent contributions [3, 8, 9]. In line with this notion, a long-acting fatty acylated GIP (acyl-GIP) was recently shown to decrease body weight and food intake by acting on the CNS GIPR [10]. And while the GIPR:GLP-1R co-agonist MAR709 decreased body weight with superior potency over a pharmacokinetically-matched GLP-1 control in wildtype mice, the superiority of MAR709 over GLP-1 vanished in mice with neuronal loss of GIPR [10]. In addition, GLP-1/GIP co-agonists lowered fasting cholesterol and triglyceride levels more efficiently than comparable benchmarked GLP-1 mono-agonist treatments in phase 2 clinical trials with T2D patients [8, 9].

Comprised of anatomically distinct depots, white adipose tissue is essential for lipid deposition. Fat accumulation in subcutaneous fat harbors little to no risk to develop metabolic complications, whereas expansion of visceral depots predisposes to the metabolic syndrome [11]. In WAT, GIPR is expressed in macrophages, pericytes endothelial and mesothelial cells. GIPR signaling enhances fat tissue blood flow, lipoprotein lipase activity, insulin action, glucose and fatty acid uptake, de novo lipogenesis and lipolysis. GIP also modulates macrophage-dependent inflammation in WAT [6].

The pharmacological potential of GIPR mono-agonism to improve systemic lipid metabolism and to reduce CVD-risk has not been fully explored yet. Particularly, it is unclear whether GIP reduces hypercholesterolemia and atherosclerotic plaque formation independent of its ability to decrease body weight and hyperglycemia. Herein we tested whether a body weight neutral dose of a previously published long acting acylated GIP analog (acyl-GIP) improves dyslipidemia and atherogenesis in male LDL receptor knock out (LDLR-/-) mice.

## Materials and methods

### Animals and diet

LDLR-/- male mice were purchased from Jackson Laboratories (https://www.jax.org/strain/002207; ME, USA) and double-housed and maintained at 22+/-2 °C, 55 +/- 10% relative humidity, and a 12-h light/dark cycle with free access to food and water. Mice were randomly assigned to treatment groups matched for body weight and fat mass. All procedures were approved by the local Animal Use and Care Committee and the local authorities of Upper Bavaria, Germany in accordance with European and German animal welfare regulations.

### Compound synthesis

The synthesis, purification, and characterization of the fatty-acylated GIP mono-agonist acyl-GIP was described previously and was used without any further chemical modification or change in formulation [3].

### Rodent pharmacological and metabolic studies

8-week old male LDLR-/- mice were fed a western diet high in calories and cholesterol (21% fat, 0.2% cholesterol, SNIFF, Germany) for 8 weeks to induce atherogenic dyslipidemia prior treatment start and were maintained on this diet during daily subcutaneous injections of vehicle or acyl-GIP (10nmol/kg body weight) in the middle of the light phase. Body weight and food intake was measured daily. Whole-body composition (fat and lean mass) was measured via nuclear magnetic resonance technology (EchoMRI, TX, USA). Fasting blood glucose and intraperitoneal glucose tolerance test (ipGTT) were determined after a 6 h-fast and 20 h after the last acyl-GIP injection. For ipGTT, 6-h fasted animals were injected intraperitoneally with 2 g glucose per kg body weight. Blood glucose was subsequently measured at time points 0, 15, 30, 60, and 120 min using a handheld glucometer (FreeStyle) as described previously [12].

### Biochemical analysis

Tail blood prior ipGTT was collected after a 6 h fast using EDTA-coated microvette tubes (Sarstedt, Germany) and immediately chilled on ice. Mice were euthanized using CO_2_ after a 4 h fast and at least 16 h after the last vehicle or compound injection. Sac blood was mixed with EDTA and immediately kept on ice. Plasma was separated by centrifugation at 5000 g at 4 °C for 10 min. Plasma levels of insulin (Crystal Chem, IL, USA), cholesterol (Thermo Fisher Scientific, MA, USA) and triglycerides (Wako Chemicals, Germany) were measured according to the manufacturers’ instructions. For lipoprotein separation, samples were pooled and analyzed via fast-performance liquid chromatography gel filtration as described previously [13].

### Histology

Atherosclerotic lesion size was assessed by analyzing cryosections of the aortic root by staining for lipid depositions with Oil-Red-O as described by previously [14]. In brief, hearts with the aortic root were embedded in Tissue-Tek O.C.T. compound (Sakura Finetek USA Inc, CA, USA) for cryosectioning. Oil-Red-O + atherosclerotic lesions were quantified in 4 μm transverse sections and averages were calculated from 3 sections. The thoraco-abdominal aorta was fixed with 4% paraformaldehyde and opened longitudinally, mounted on glass slides and stained enface with Oil-Red-O. Aortic arches with the main branch points (brachiocephalic artery, left subclavian artery and left common carotid artery) were fixed with 4% paraformaldehyde and embedded in paraffin. Lesion size was quantified after Hematoxylin and Eosin (H&E)-staining of 4 μm transverse sections and averages were calculated from 3 to 4 sections.

### RNA sequencing

Total RNA was extracted from liver, inguinal (subcutaneous) and gonadal (visceral) white adipose tissue (iWAT (n = 4/treatment) and gWAT (n = 5/treatment), respectively of vehicle and Acyl-GIP vehicle treated LDLR-/- mice (n = 5) using Qiazol according to the manufacturer’s instructions (Qiazol Lysis Reagent, QIAGEN, Germany). The quality of the RNA was determined with the Agilent 2100 BioAnalyzer (RNA 6000 Nano Kit, Agilent, CA, USA). All samples with an RNA integrity number (RIN) had a value greater than 7. For library preparation, 1 µg of total RNA per sample was used. RNA molecules were poly(A) selected, fragmented, and reverse transcribed with the Elute, Prime, Fragment Mix (EPF, Illumina, CA, USA). End repair, A-tailing, adaptor ligation, and library enrichment were performed as described in the TruSeq Stranded mRNA Sample Preparation Guide (Illumina, CA, USA). RNA libraries were assessed for quality and quantity with the Agilent 2100 BioAnalyzer and the Quant-iT Pico-Green dsDNA Assay Kit (Life Technologies, CA, USA). Strand-specific RNA libraries were sequenced as 150 bp paired-end runs on an Illumina HiSeq4000 platform. The STAR aligner* (v 2.4.2a)57 with modified parameter settings (–twopassMode = Basic) was used for split-read alignment against the mouse genome assembly mm10 (GRCm38) and UCSC known Gene annotation. To quantify the number of reads mapping to annotated genes we used HTseq-count (v0.6.0). For differentially testing we followed guidelines reported by Law et al. [15]. Briefly, we excluded genes with zero counts in all samples and further removed genes with cumulative counts per million in less than five samples. We used the edgeR package for data pre-processing, followed by the limma package with its voom method, linear modelling and empirical Bayes moderation to assess differential expression. We used EnrichR web interface for gene and pathway enrichment. As input, genes with a p-value < 0.05 and a logFC > 0.75 were used.

### Statistics

Statistical analyses were performed using GraphPad Prism8. The Kolmogorov-Smirnov test was used to assess for normality of residuals. The unpaired Student two-tailed t-test was used to detect significant differences. A Grubbs test (α < 0.05) was used to detect significant outliers, which were then excluded from subsequent statistical analysis and figure drawing. P < 0.05 was considered statistically significant. All results are mean ± SEM unless otherwise indicated.

## Results

### GIPR-agonist acyl-GIP ameliorates dyslipidemia and atherosclerotic plaque formation in male LDLR-/- mice independently of weight loss

**Fig. 1:**
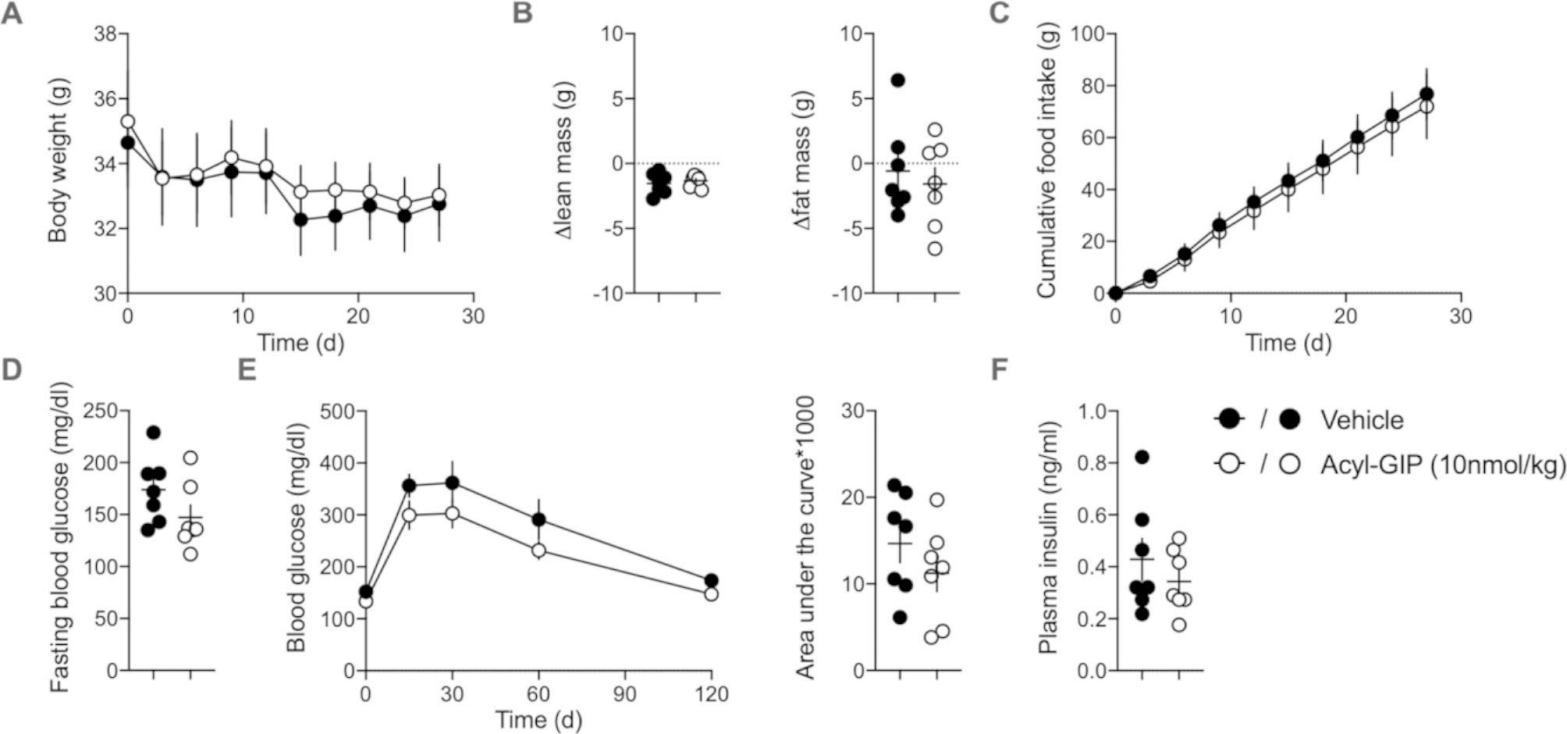
Body weight neutral dose of acyl-GIP in LDLR-/- mice. (**A**) Body weight, (**B**) change of body composition, (**C**) cumulative food intake, (**D**) fasting blood glucose, (**E**) interperitoneal glucose tolerance and (**F**) fasting insulin levels of male LDLR-/- mice treated daily with either vehicle or acyl-GIP via subcutaneous injections for 27 days. n = 7. Except for ipGTT (performed at study day 21), plasma parameters were measured from sac blood at study end (day 27). Data represent means ± SEM

We used LDLR-/- mice to test chronic acyl-GIP treatment in a mouse model of dyslipidemia-induced atherosclerosis. Recently, Mroz et al. showed that optimized long-acting GIP peptide analogs reduce body weight of diet-induced obese (DIO) mice [16]. To assess whether acyl-GIP affects lipid metabolism and atherosclerotic plaque formation independently of weight loss, we used a dose of acyl-GIP (10 nmol/kg) that is subthreshold for reducing body weight and for improving glucose metabolism. Consistent with this, body weight (Fig. 1A), body composition (Fig. 1B) and food intake (Fig. 1C) remained similar between vehicle and acyl-GIP treated LDLR-/- mice. Glucose metabolism of LDLR-/- mice was only marginally impaired and could not be improved further by acyl-GIP (Fig. 1D-E). Fasting insulin levels were similar between vehicle and acyl-GIP treated mice at study end (Fig. 1F).

**Fig. 2:**
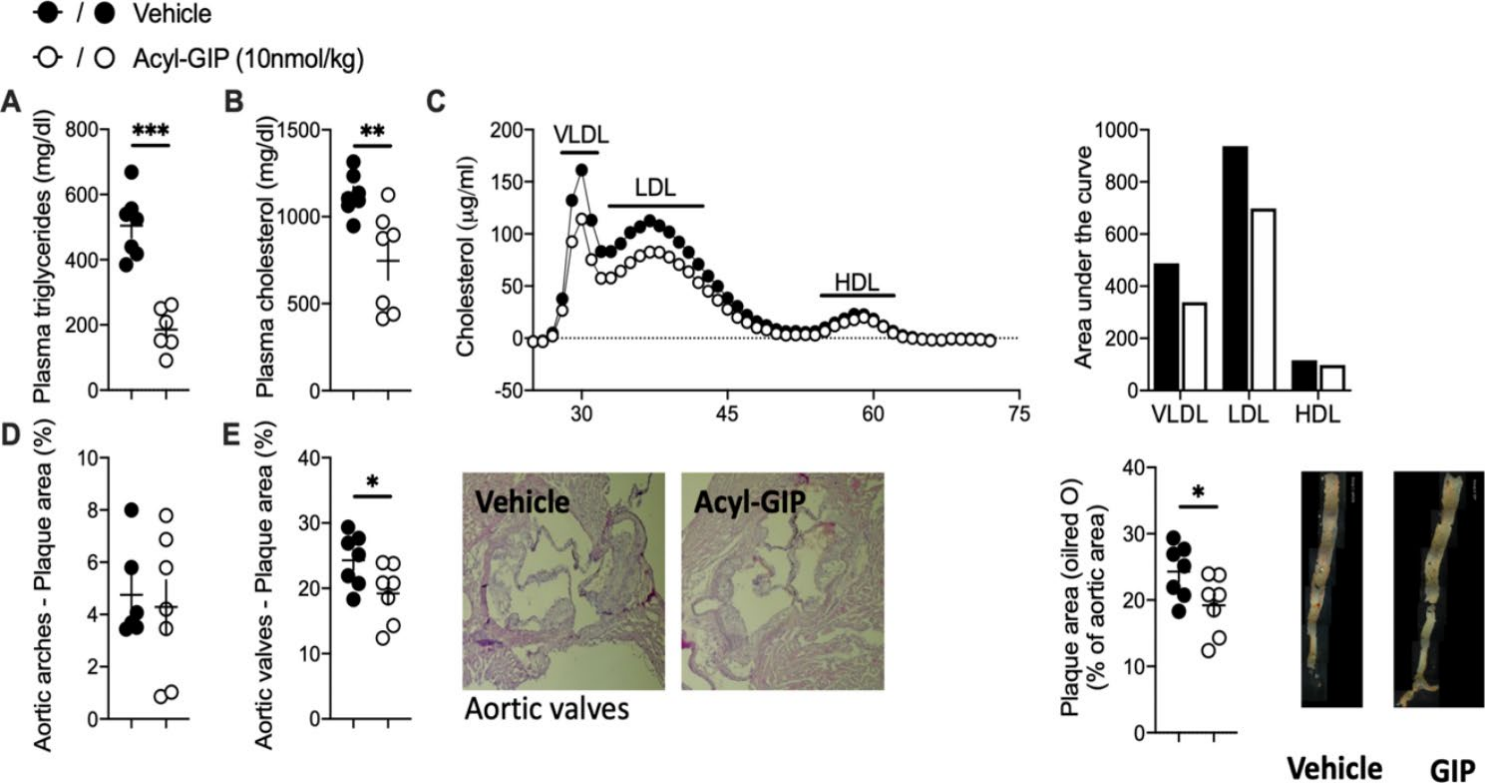
Acyl-GIP ameliorates dyslipidemia and atherosclerotic plaque formation in LDLR-/- male mice. Plasma (**A**) triglycerides, (**B**) cholesterol and (**C**) lipoprotein fractions as well as (**D** and **E**) the percentage of plaque area in aortic arches and valves and along the descending aorta of male LDLR-/- mice treated daily with either vehicle or acyl-GIP via subcutaneous injections for 28 days. n = 7. Blood lipids were determined from sac plasma at the end of the study. Data represent means ± SEM. *P < 0.05, **P < 0.01, *** P < 0.001, determined by unpaired two-sided t-test

Acyl-GIP treatment for 28 days remarkably reduced fasting plasma triglycerides and total cholesterol levels in LDLR^−/−^ mice (Fig. 2A-B). This GIP-mediated reduction of plasma lipid levels was mainly attributable to a decrease of the VLDL and LDL lipoprotein fractions, while HDL levels remained similar to vehicle treated mice (Fig. 2C). Acyl-GIP induced alterations in lipid metabolism were thus independent of changes in glucose metabolism. Similarly to human trials [17], we observed that the acyl-GIP-mediated improvement of dyslipidemia in LDLR-/- mice was independent of changes in insulin metabolism. Assessing effects of chronic acyl-GIP treatment on liver metabolism we found that liver weights were not altered in acyl-GIP treated mice compared to vehicle treated mice (Figure S1A) and that GIPR was only marginally expressed in hepatic tissue (Figure S1B), indicating an indirect effect of acyl-GIP treatment on liver metabolism. RNAsequencing analysis in livers of chronically acyl-GIP treated mice revealed significantly altered gene expression changes compared to vehicle treated mice (Figure S1C). In line with decreased plasma lipids, genes associated with cholesterol and triglyceride metabolism were among the most significantly down regulated pathways in livers after acyl-GIP treatment (Figure S1D). Interestingly, pathways related to cholesterol biosynthesis were up regulated by acyl-GIP treatment. This may indicate that acyl-GIP-mediated cholesterol lowering stimulates the synthesis of genes involved in cholesterol biosynthesis as a feed-back mechanism. Most importantly, acyl-GIP treatment was accompanied by reduced atherosclerotic plaque formation within the aortic valve (Fig. 2G-H) and decreased fat streaks along the descending aorta (Fig. 2I).

### Acyl-GIP targets subcutaneous adipose tissue of LDLR-/- male mice

**Fig. 3:**
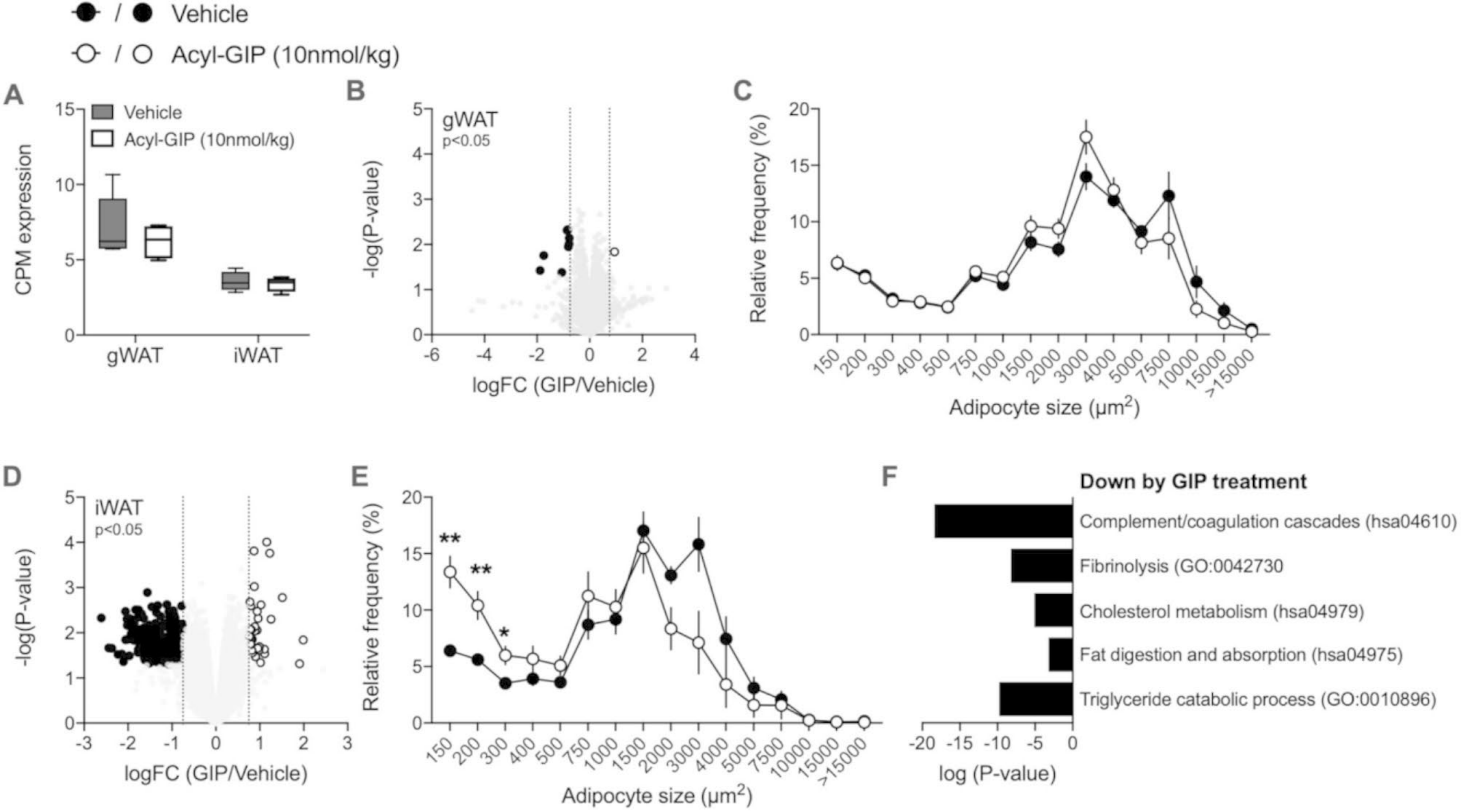
Acyl-GIP regulates adipocyte size and gene expression in subcutaneous fat of LDLR-/- mice. (**A**) Relative gWAT and iWAT GIPR gene expression of vehicle (gWAT n = 5; iWAT n = 4) and acyl-GIP treated LDLR-/- mice (gWAT n = 5; iWAT n = 4). (**B**) Volcano plot showing differential expression and its significance (-log10(p-Value), limma-trend) and (**C**) frequency distribution of adipocyte cell sizes (µm2) of gWAT from acyl-GIP (RNA sequencing n = 5; histology n = 7) versus vehicle (RNA sequencing n = 5; histology n = 6) treated LDLR-/- mice. (**D**) Volcano plot showing differential expression and its significance (-log10(p-Value), limma-trend) and (**E**) frequency distribution of adipocyte cell sizes (µm2) of iWAT from acyl-GIP (RNA sequencing n = 5; histology n = 4) versus vehicle treated LDLR-/- mice (RNA sequencing n = 5; histology n = 4). (**F**) Gene ontologies (p < 0.0001) and KEGG pathways (Pvalue < 0.001) that are down regulated by acyl-GIP in sc fat. Representative terms from Supplementary Table 2 are depicted

Based on the fact that GIP has been shown to modulate lipid disposal in adipose tissue [6], we investigated whether our long-acting acyl-GIP agonist also affects adipocyte size and gene transcription in visceral (gonadal; gWAT) and/or subcutaneous (inguinal; iWAT) of our treated LDLR-/- male mice. We performed RNA sequencing of gWAT and iWAT of LDLR-/- mice to explore treatment induced transcriptional changes at study end (Tables S1-2). Despite higher GIPR expression in gWAT compared to iWAT (Fig. 3A), acyl-GIP treatment resulted in more pronounced gene expression changes in iWAT than gWAT and reduced adipocyte size in subcutaneous but not visceral depots (Fig. 3B-E). In line with decreased plasma lipids, genes associated with cholesterol and triglyceride metabolism were among the most significantly down regulated pathways in iWAT after acyl-GIP treatment (Fig. 3F). Moreover, acyl-GIP treatment decreased the expression of genes within the complement and coagulation cascades as well as the fibrinolysis pathway (Fig. 3F). In addition, our findings that chronic acyl-GIP treatment predominately changed adipocyte size and gene transcription in targeted subcutaneous fat and to a lesser extent in visceral fat of male LDLR-/- mice may suggest a fat depot preference of our GIP-agonist. To test if these observations derive from a direct effect of acyl-GIP on fat cells or result from an indirect mechanism affecting adipocytes requires further examinations.

## Discussion

Herein we identified an unanticipated efficacy of chronic acyl-GIP administration to improve dyslipidemia and CVD in a western diet-induced mouse model of atherosclerosis independently of body weight loss, indicating a specific acyl-GIP-induced effect within the treatment spectrum of clinically advancing novel poly-pharmacological approaches for obesity and T2D. These findings might initiate future studies to explore the potential of GIP mono- or poly-pharmacology to treat disturbances of lipid metabolism, which contributes to reduced cardiovascular mortality.

Although GLP-1/GIP co-agonists are one of the most promising drugs to treat obesity and diabetes and have been shown to reduce fasting cholesterol and triglycerides in T2D patients [8, 9], GIP-dependent contributions to metabolic benefits achieved with this combinatorial therapy remain unclear. GIP plays a physiologic role in the disposition of ingested fat by stimulating lipid uptake in subcutaneous adipose tissue [17–21]. This effect is pronounced in lean individuals and blunted in obese and T2D subjects [22]. Moreover, high fasting plasma GIP levels were associated with low plasma LDL cholesterol in both, men and women, and low plasma triglycerides in women at risk for developing T2D [17]. These associations were independent of fasting plasma insulin levels. Taking into account the fact that the herein observed acyl-GIP-induced improvement of dyslipidemia in LDLR-/- mice was also independent of changes in plasma insulin levels points to a direct effect of acyl-GIP on adipocyte metabolism. In addition, our findings that chronic acyl-GIP treatment predominately targeted subcutaneous fat and to a lesser extent visceral fat in male LDLR-/- mice suggests a fat depot preference of our GIP-agonist. Of note, our RNAseq analysis indicated that pathways such as the complement and coagulation cascade or fibrinolysis were significantly down regulated by acyl-GIP compared to vehicle treatment in western diet fed male LDLR-/- mice. These findings are of interest as alterations in the hemostatic system are associated with WAT dysfunction and the prothrombotic state observed in obesity [23] and thus may suggest an ulterior acyl-GIP-mediated effect in adipose tissue. It should be mentioned at this point that higher fasting GIP levels have been reported in correlation with an unhealthy fat distribution as indicated by a higher visceral to subcutaneous fat distribution exclusively in men, but not women [17]. Thus, potential sex-specific differences of GIP action on visceral and subcutaneous adipose tissue physiology warrants further examination.

Interestingly, there is evidence in the literature that body weight loss by caloric restriction re-sensitized obese individuals to GIP action in subcutaneous fat [24]. Hence, one can assume that GLP-1/GIP mediated weight loss could actually prime GIP action to improve dyslipidemia.

It is very difficult to assess GIPR receptor occupancy by acyl-GIP, also because it would be different based on which tissue is under examination. The herein used acyl-GIP requires 60–100 nmol/kg to affect body weight and food intake in diet induced obese rodents [10]. Thus, the applied dose of 10 nmol/kg was hence clearly subthreshold to affect body weight, food intake and also glycemia. Together with the known effect of GIP to regulate lipid metabolism in adipocytes [25] our findings might initiate future studies to explore the potential of GIP mono- or poly-pharmacology to treat disturbances of lipid metabolism and potentially reducing cardiovascular mortality. It is important here to state that our findings have been observed in a rodent model for cardio-metabolic disease and thus it is impossible at this point to extrapolate to humans without further investigations. It is important to note that disorders in triglyceride and cholesterol metabolism are major risk factors for the development of lethal atherosclerotic cardiovascular complications in obese individuals and T2D patients. Besides body weight and glucose management, this is particularly relevant in light of the recent consensus in the field that the growing prevalence of cardio-metabolic disease will perhaps be the greatest health challenge throughout the world and that therefore multifaceted interventions and treatments in a new era of precision medicine will be required to provide the best possible comprehensive care for patients with cardiometabolic disease [26–28].

We just recently showed that Tirzepatide is only a weak and partial agonist at the mouse GIPR with a 75-fold less potency at the mouse relative to human GIPR [29]. Based on these findings it seems plausible that Tirzepatide is not suitable to assess the mode of action of GIPR agonism and GIPR:GLP-1R co-agonism in mice and was hence omitted herein.

Regarding future obesity treatment strategies implementing novel GIP/GPL-1 co-agonists that are emerging it is unclear whether every co-agonist will be as beneficial as and superior to single GLP-1R agonism. For example, the metabolic effect of NNC0090-2746 relative to liraglutide has been tested at a single dose for only 12-wks of treatment [8]. This study design seems suboptimal in many different aspects due to the lack of multiple higher doses which are crucial for e.g. Tirzepatide to maximize weight loss. In addition, the study duration of 12 wks may not have been long enough in light of the SURPASS trials showing that much longer treatment durations are required to see the maximal effects on weight loss and improvement in glucose control.

## Conclusions

GLP-1/GIP co-agonists are one of the most promising drugs to treat obesity and diabetes and have been shown to reduce fasting cholesterol and triglycerides in T2D patients. Here we show that the long-acting GIP mono-agonist acyl-GIP reduced hyperlipidemia and atherosclerotic lesion formation in male LDLR-/- mice independently of body weight loss indicating an effect exclusively mediated by GIP signaling. Mono-agonistic treatment with acyl-GIP may thus be a novel approach to alleviate cardiometabolic disease without changing body composition.

## Electronic supplementary material

Below is the link to the electronic supplementary material.

Supplementary Material 1

Supplementary Material 2

Supplementary Material 3

## Data Availability

The datasets used and/or analysed during the current study are available from the corresponding author on reasonable request.

## List of Abbreviations

GIP: Glucose-dependent insulinotropic polypeptide
GIPR: Glucose-dependent insulinotropic polypeptide receptor
GLP-1: Glucagon-like peptide-1
GLP-1R: Glucagon-like peptide-1 receptor
LDLR: Low density lipoprotein receptor
VLDL: Very low density lipoprotein
HDL: High density lipoprotein
Acyl-GIP: Acylated GIP analog
ipGTT: Intraperitoneal glucose tolerance test
CVD: Cardiovascular disease
WAT: White adipose tissue
iWAT: Inguinal WAT
gWAT: Gonadal WAT
DIO: Diet-induced obese
T2D: Type 2 Diabetes
