# Correction: GIP receptor agonism improves dyslipidemia and atherosclerosis independently of body weight loss in preclinical mouse model for cardio-metabolic disease

**DOI:** 10.1186/s12933-024-02407-8

**Published:** 2024-09-12

**Authors:** Stephan Sachs, Anna Götz, Brian Finan, Annette Feuchtinger, Richard D. DiMarchi, Yvonne Döring, Christian Weber, Matthias H. Tschöp, Timo D. Müller, Susanna M. Hofmann

**Affiliations:** 1 Institute for Diabetes and Regeneration, Helmholtz Diabetes Center at Helmholtz Zentrum München, German Research Center for Environmental Health (GmbH), 85764 Neuherberg, Germany; 2 Institute for Diabetes and Obesity, Division of Metabolic Diseases, Helmholtz Diabetes Center at Helmholtz Centre Munich, Munich, Germany; 3 Technische Universität München, 80333 Munich, Germany; 4 Novo Nordisk Research Center Indianapolis, Indianapolis, IN USA; 5 Research Unit Analytical Pathology, Helmholtz Center Munich, 85764 Neuherberg, Germany; 6 Department of Chemistry, Indiana University, Bloomington, IN USA; 7 Department of Angiology, Swiss Cardiovascular Center, Inselspital, Bern University Hospital, University of Bern, Bern, Switzerland; 8 Institute for Cardiovascular Prevention (IPEK), Ludwig-Maximilians-University Munich, Munich, Germany; 9 DZHK (German Centre for Cardiovascular Research), Partner Site Munich Heart Alliance, Munich, Germany; 10 Department of Biochemistry, Cardiovascular Research Institute Maastricht (CARIM), Maastricht University, Maastricht, the Netherlands; 11 Munich Cluster for Systems Neurology (SyNergy), Munich, Germany; 12 German Center for Diabetes Research (DZD), 85764 Neuherberg, Germany; 13 Department of Medicine IV, University Hospital, LMU Munich, Munich, Germany

**Correction to: Cardiovasc Diabetol (2023) 22:217**

10.1186/s12933-023-01940-2

Following publication of the original article [1], the author noticed the errors in Fig. 2 and in Results section.

The bar graph is mistakenly duplicated in “percentage of plaque area of the aortic valves” of Fig. 2E. The corrected figure is given below:

**Fig. 2:**
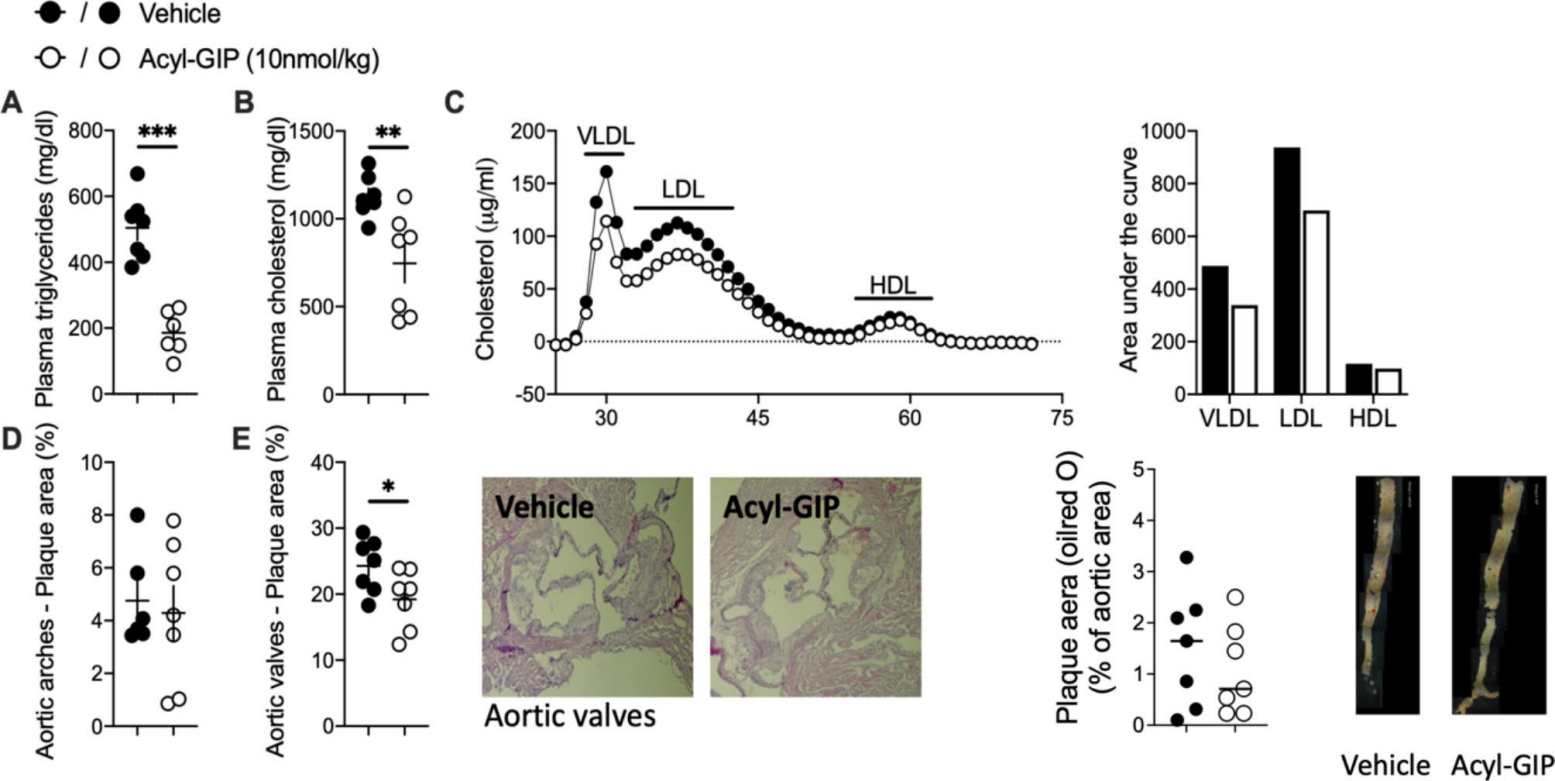
Acyl-GIP ameliorates dyslipidemia and atherosclerotic plaque formation in LDLR-/- male mice. Plasma (**A**) triglycerides, (**B**) cholesterol and (**C**) lipoprotein fractions as well as (**D** and **E**) the percentage of plaque area in aortic arches and valves and along the descending aorta of male LDLR-/- mice treated daily with either vehicle or acyl-GIP via subcutaneous injections for 28 days. *n* = 7. Blood lipids were determined from sac plasma at the end of the study. Data represent means ± SEM. *P < 0.05, **P < 0.01, *** *P* < 0.001, determined by unpaired two-sided t-test

In Result section under the heading “GIPR-agonist acyl-GIP ameliorates dyslipidemia and atherosclerotic plaque formation in male LDLR-/- mice independently of weight loss”, the last sentence should read “Most importantly, acyl-GIP treatment was accompanied by reduced atherosclerotic plaque formation within the aortic valve and a trend to decrease fat streaks along the descending aorta (Fig. 2E)“ instead of “Most importantly, acyl-GIP treatment was accompanied by reduced atherosclerotic plaque formation within the aortic valve (Fig. 2G–H) and decreased fat streaks along the descending aorta (Fig. 2I)”.
