# Supplementary material for: GIP receptor agonism improves dyslipidemia and atherosclerosis independently of body weight loss in preclinical mouse model for cardio-metabolic disease
Source: Cardiovasc Diabetol. 2023 Aug 17;22:217. doi: 10.1186/s12933-023-01940-2 (PMC10436634; doi:10.1186/s12933-023-01940-2)
Supplement: Supplementary file 3 — Supplementary Material 3 [file 12933_2023_1940_MOESM3_ESM.pdf]

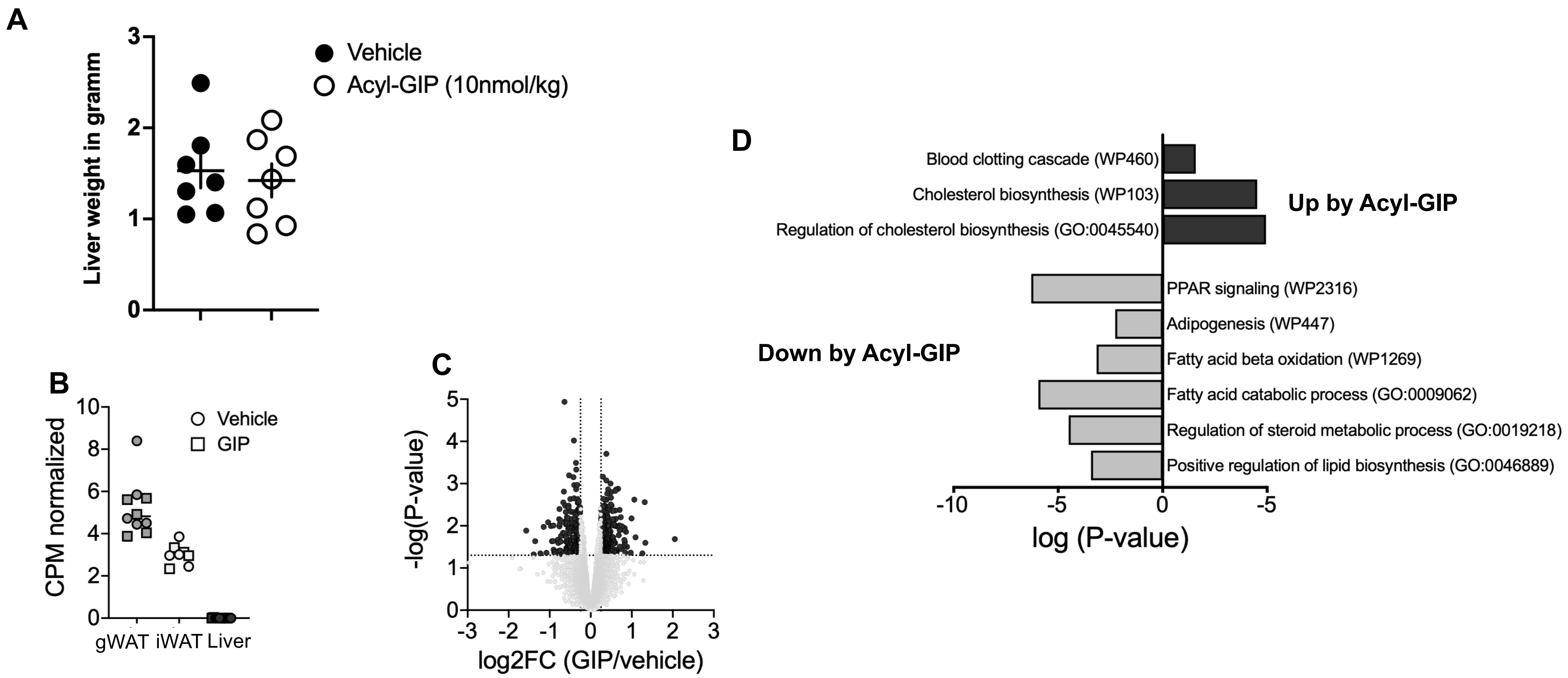

Figure S1: (A) Liver weights of vehicle and Acyl-GIP treated male LDLR-/- mice. (B) Relative gWAT, iWAT and liver GIPR gene expression of vehicle and Acyl-GIP treated LDLR-/- mice (gWAT n=5; iWAT n=4; liver n= 4). (C) Volcano plot showing differential expression and its significance (-log10(p-Value), limma-trend) of chronic Acyl-GIP effects on liver. (D) Pathway regulation in livers of male LDLR-/- mice after Acyl-GIP treatment compared to vehicle treatment.
